# Suicide

**Published:** 1823-06-01

**Authors:** 


### Medico-Chirurgical Review,

AND

JOURNAL OF MEDICAL SCIENCE.

(Analytical Series.)

"Nec Aranearum textus ideò melior, quia ex se fila fingunt; nec noster vilior, quia ex alienis libamus, ut apes."

Vol. IV.] JUNE 1, 1823. [No. 13.

### I.
### SUICIDE.

1. *Du Suicide.* Par M. Esquirol. Dict. des Sciences Med. Vol. LIII.

2. *Du Suicide.* Par J. P. Falret, M. D. Octavo pp. 358. Paris, 1822.

Nothing can be more true than that "self-preservation is the first law of Nature," from the lord of the creation down to the minutest insect. How is it then, that man alone lifts his hand against his own life? Various motives lead him to this unnatural act. But, however various may be the causes, they almost all and always tend to over-excite the imagination, and lead it to dwell on some real or fancied good more precious than life—or some real or fancied ill more formidable than death. An examination of the principal circumstances or causes, which lead to self-destruction, will convince us that this act does not always deserve the name of suicide—or at least that idea of reproach connected with self-murder. Neither can this act be considered as *always*, though very generally, the effect of mental alienation. Victims to notions false but accredited—to customs barbarous yet national, not only individuals but whole sects have devoted themselves to voluntary death. Almost every passion has its tide, which rises occasionally to an excess that sacrifices all things that oppose it—not even excepting the life of the individual. In febrile delirium, in insanity, suicide is more frequently committed than people imagine. Hypochondriasis and lypemania not unfrequently lead to that hatred and ennui of life which ends in self-murder.

We have said that self-destruction cannot always be classed with, or denominated culpable or maniacal suicide. He who nobly and generously exposes his own life to inevitable destruction, in obedience to laws or religious creeds, or to preserve inviolate his honour or his oath, cannot surely be stiled a self-murderer. Such was Codrus, who sought death in the enemy's camp to accomplish the oracle that promised victory to the Athenians—such were the generous souls of Calais and Rouen, who offered themselves a sacrifice to save the lives of their fellow citizens ready to perish by famine and the sword of the enemy. Were Socrates and Regulus suicides because one swallowed hemlock rather than violate the laws, and the other returned to Carthage rather than forfeit his word of honour? Are we to give the name of suicide to those enthusiasts who are taught by their religion and incited by their priests to devote themselves to voluntary death, as an act the most honourable in this world, and sure to be rewarded in a world to come? Instances of this kind were numerous among the Thracians, ancient Germans, Arabs—and even now among the Hindoos. By the Gymnosophists life was taught to be despised, and death represented as the summum bonum. Disease, infirmity, old age, among them were opprobria—and natural death dishonorable. In the capital of the Isle of Cos, the native land of Simonides, no septuaginarians were to be seen. Suicide was permitted by the laws after the age of 60 years, and it was generally performed amidst the assembled relations, by drinking a deadly potion of poppy or hemlock. It would be endless to relate the various customs of this kind which have prevailed in different countries. The burning of the Hindoo widows, and the voluntary sacrifices annually made under the wheels of the Jaggernaut car, are modern examples of fanatical suicide.

1. *Etiology*. Among the predispositions to this dreadful act, Dr. Falret includes the hereditary. Suicidal melancholia he reckons as one of the species of mental alienation, which is most readily transmissible from parent to progeny. This is confirmed by the testimony of Esquirol, Spurzheim, Rush, and even Voltaire. Dr. F. relates several instances in illustration of this hereditary disposition; among the rest the celebrated Barthez, whose father starved himself to death at the age of 90 years, in consequence of the loss of his second wife; while the son was haunted with a similar propensity in his old age, on account of the death of a favourite servant. He lamented, however, that he had not courage to put the father's example into practice.

*Temperament*, M. Falret thinks, has no small influence in the production of the suicidal tendency. He instances the unhappy fate of our countryman Chatterton, the Bristol poet, who, a few days before his death, (by arsenic) wrote to his mother in these terms:—"I am about to quit, for ever, my ungrateful country. I shall exchange it for the deserts of Africa, where tygers are a thousand times more merciful than man!" This unfortunate youth, we need hardly say, was nearly dead with hunger in this ungrateful land of luxury, when he swallowed poison to end his misery! The offsprings of his fine genius were so ill judged, and badly paid, that he could not procure the common necessaries of life!

The sanguine temperament is favourable to the development of the suicidal tendency, in consequence of the irascibility, excessive sensibility, and natural impetuosity of this class of people. In respect to age, we observe less of the suicidal disposition in youth and senectitude, than in adolescence. The reason is evident enough. It is at this latter period that the passions burst forth in all their impetuosity, and render man too prodigal of life. In old age man becomes as parsimonious of his life as of his purse. Nevertheless we have examples of suicide in the two extremes of existence. M. Falret knew a boy of twelve years of age hang himself because he could not get higher than the twelfth in his class. A melancholy example of this kind lately occurred in Westminster School, where a fine boy threw himself out of a window in consequence of some chagrin of mind. Dr. F. relates the case of a gentleman, 75 years of age, who hanged himself on the 17th July, 1819, in Paris. A paper was found in his desk, written with his own hand, and containing these words:—"Christ has said that, when the tree is old, and incapable of bringing forth fruit, it is to be hewn down and thrown into the fire."

Females, who are much more disposed to melancholy than men, are yet much less addicted to suicide.* This is probably to be attributed to their comparative weakness of physical constitution, to their gentleness of character—but still more, we think, to their patience and resignation under the dispensations of Providence, and under the calamities and crosses of life.

To the vicious modes of education—to the seduction of novel-reading—to the effeminating effects of music, and of theatrical representations, our authors (M. Falret in particular) attach much influence in generating a suicidal tendency. These, we apprehend, can only act by corrupting

* According to the experience of Falret and Esquirol, the comparative frequency among males and females is as three of the former to one of the latter.

the morals and deteriorating the health—and thus far predisposing to various other crimes and casualties in common with suicide.

The effects of climate have been greatly exaggerated. The climate of England, for instance, must be nearly the same now as at the Roman invasion, when suicide was unknown in this land. So must have been the climate of Italy; yet suicide was common among the Romans, and is now rare among the Italians. It is to moral and physical circumstances, of course, that we are to look for the principal causes of these revolutions, rather than to climate. Did the battle of Pharsalia change the climate of Italy, when it produced so many suicidal acts? At Copenhagen the number of suicides have doubled within the last ten or twelve years—without any change of climate. The same may be said of France after the revolution. At the same time we must not assert that climate has no influential effect. A gloomy atmosphere, like ours, exasperates the nervous and melancholic temperament, and may thus conduce indirectly to self-destruction.

Onanism is considered by many writers as tending to this crime—and so is a life of idleness succeeding activity. For obvious reasons suicide is infinitely more prevalent among the rich and profligate, than among the poor and consequently industrious classes of society.

But among all classes it is to the *passions* we must look for the paramount cause of suicidal attempts. We all know that when passion rises to a certain extent, the whole physical and intellectual economy of man is disordered. The organic functions are, as it were, perverted, as well as the reasoning faculty—in short, a man under the influence of unbridled passion (of whatever kind) is in a state of temporary insanity, and then commits actions diametrically opposed to his real interests and natural dispositions. Thus terror will often deprive us of the thought of flight, and plunge us into greater danger than that by which we are menaced—love, in excess, deprives the individual of the powers or qualifications necessary for the accomplishment of his object—jealousy will drive a man of the most amiable disposition to imbrue his hands in the blood of his best friend!

The suicidal tendency generated by the passions is either acute or chronic. Under a gust or ebullition of passion a man may quickly destroy himself—but more frequently it is of a chronic nature, and the rankling passion gives occasion to a premeditated act. "Yet," says Esquirol, "it is certain that, up to the moment of execution, the man who attempts his own life is almost always like a man in a state of despair or intellectual aberration." ("Semblable presque toujours à un homme désesperé et dans le delire.")

Dr. Falret places the passion of love at the head of the list of those which most powerfully derange both body and mind. We do not consider this passion as by any means the most common in the production of suicide. Blighted ambition; the loss of fortune by gambling; scepticism; and religious melancholy, we apprehend, are far more frequently the causes of self-murder than erotomania. Love, however, is by no means an infrequent cause of this dreadful catastrophe. Dr. Falret relates the case of a young apothecary, who, on receiving a rebuff from his sweetheart, went home and blew out his brains, having first written the following sentence on his door. "When a man knows not how to please his mistress, he ought to know how to die." Esquirol was called to a lady who refused to take any sustenance in consequence of surprizing her husband in an improper situation with another woman. She died on the seventh day, notwithstanding every effort to divert her from her fixed and desperate resolution.

The sense of shame or dishonour is a very powerful impulse to suicide, as attested by all history. Lucretia could not survive the outrage of Tarquinius Sextus, and therefore plunged a dagger in her breast. The Roman generals, in the civil wars, usually committed suicide when unsuccessful in battle, to prevent the mortification of coming under the yoke. These classical suicides, if we may use such an expression, are rendered familiar to us all by the histories and tragedies of Brutus and Cassius, Anthony and Cleopatra, Cato, and other celebrated characters constantly represented on our stages.*

"Insensibly undermined," says Dr. Esquirol, "by the effects of hatred, of jealousy, of blighted ambition, of squandered fortune, man

---

* Remorse has doubtless wrought many a self-murder. M. Guillon, in his memoirs of suicides, relates the following remarkable instance. The Chevalier de S——— had had seventeen affairs of honour, in each of which his adversary fell. But the images of his murdered rivals began to haunt him night and day; and at length he fancied he heard nothing but the wailings and upbraidings of seventeen families—one demanding a father, another a son, another a brother, another a husband, &c. Harrassed by these imaginary followers, he shut himself up in the monastery of La Trappe; but the French revolution threw open this asylum, and turned the Chevalier once more into the world. He was now no longer able to bear the remorse of his own conscience, or, as he supposed, the sight of seventeen murdered men, and therefore put himself to death. It is evident that insanity was here the consequence of the remorse and the cause of the suicide.

is brought slowly, and by successive paroxysms, to the most dreadful resolutions. But acting thus slowly, the passions act not the less effectually in enfeebling the physical powers and disordering the intellectual faculties of man. Many unfortunate people, under such circumstances, have attempted their own lives, while they scarcely knew what they were doing. Many have assured me that they could remember nothing of the transaction."

*Physical* pain or suffering, which leads often to hypochondriasis and even insanity, is sometimes productive of suicide —but much less frequently than *mental* anguish.

"He, (says M. Esquirol,) who has no intervals of ease from corporeal pain—who sees no prospect of relief from his cruel malady, fails, at length, in resignation, and destroys his life in order to put a period to his sufferings. He calculates that the pain of dying is but momentary, and commits the act in a cool and meditated despair. It is the same in respect to *moral* condition, that drives the hypochondriac to suicide, who is firmly persuaded that his sufferings are beyond imagining—that they are irremediable, either from some fatal peculiarity in his own constitution, or the ignorance of his physicians. It is a remarkable feature in hypochondriasis, that in no other disease, is there such a fear of death and a desire to die combined!—Both fears proceed from the same pusillanimity. Finally, it may be remarked, that the hypochondriac talks much of death—often wishes his attendants to perform the friendly office—even makes attempts on his own life—but rarely accomplishes the act. The most trifling motive, the most frivolous pretext, is a sufficient excuse for procrastinating, from day to day, the threatened catastrophe."*

We have said in another part of this Journal, that *physical* pain but rarely leads to suicide. We still adhere to this assertion, particularly as it regards the female sex. Nevertheless, we do not mean to deny that corporeal suffering occasionally drives its victim to this dreadful mode of terminating bodily anguish. But, we apprehend, that the intellect generally suffers prior to this finale. Licinius Cœcinius, the pretor, subdued by the pain and ennui of a tedious disease,

* Dr. Falret relates a melancholy case that happened lately in a provincial mad-house in France. An apothecary was there confined, who was haunted with the ennui and hatred of life, and who was always begging his companions to put him to death. At length, an insane patient was admitted who instantly complied with the apothecary's request. They watched an opportunity, got out of a window into the back yard, and from thence into the kitchen. They pitched upon the cook's chopper, and the apothecary laying his neck on a block, his companion deliberately and effectually severed the head from the body. He was seized and examined before a tribunal, where he candidly confessed the whole transaction, and observed that he would again perform the same friendly office for any unhappy wretch who was tired of his existence!

swallowed opium. Haslam relates the case of a gentleman, who destroyed himself to avoid the tortures of gout.* Esquirol knew a young lady who, by a diabolical attempt made on her virtue, was thrown into convulsions, which frequently returned and rendered her life distressing. She made reiterated attempts to poison herself, but always failed, and after several years of great suffering recovered.† The pellagra of Italy occasionally produces suicide, but is then generally attended with symptoms denoting considerable determination to the head.

The loss of beauty, by disease or age, has, we believe, more frequently driven females to self-murder, than any of those painful maladies, as cancer, &c. to which they are peculiarly liable. Georget and Falret have each related some curious cases of this kind.

Dr. Esquirol remarks, that maniacs often commit suicide, where reflection has little or no concern in the act. They generally precipitate themselves from a height, which shews that they obey a blind impulse, and employ a mean the most easy of execution, and least liable to interruption. The insane, he observes, live under the constant influence of illusions, pursued by imaginary dangers, the sport of their hallucinations. They are often destroyed by false calculations, when they are supposed to have committed deliberate suicide. Thus, it is not uncommon for a lunatic to open a window and fall down into the area, supposing himself going into his apartment. A maniac who was harrassed with bulimia died suddenly; and, on examination, it was found that he had swallowed a sponge, in a mistake for some kind of food, which had stuck in the œsophagus and compressed the windpipe.

"A monomaniac," says Esquirol, "hears a voice within him, which repeats these words—'*kill thyself, kill thyself.*' He, therefore, commits suicide in obedience to this superior power, whose order he

---

* It is recorded, that the pain of gout drove Servius, the grammarian, to death by poison. Pliny the younger informs us, that one of his friends, Cornelius Rufus, having in vain sought remedies to palliate the tortures of this disease, starved himself to death at the age of 67 years. It is related of Pomponius Atticus, and the philosopher Cleanthes, that they both starved themselves to death, in order to get rid of physical pain. In the course of these attempts, the corporeal sufferings were removed—probably in consequence of the great exhaustion and attenuation—but both individuals persevered till death took place, observing that, as this final ordeal must one day be undergone, they would not now retrace their steps or give up the undertaking. These instances corroborate our opinion, that pain deranges the intellect before the individual determines on suicide.

† M. Esquirol. Dict. des Sciences Med. Vol. LIII. p. 219.

dare not withstand. A man under a religious hallucination believed himself in communication with the Deity. He hears a celestial voice saying;—'*my son, come and seat thyself by my side.*' He opens the window to obey the invitation, falls down, and fractures his leg. When carried to his bed, he expresses the greatest astonishment how he could possibly have come by the fall and the wound."

Dr. Esquirol relates a great many curious instances of maniacal suicide, shewing the intimate connexion between disordered intellect and tendency to self-murder. A very remarkable case was that of a gentleman, of some political consequence, who had an attack of apoplexy, from which he recovered by copious blood-letting. Some years afterwards he had a fall from his horse, and was wounded severely in the head, the injury occasioning fever and delirium of some weeks' duration. After this accident, he evinced some marks of mental aberration. He threw up his post under government, and retired to his chateau in the country, for the purpose of maturing a scheme for *uniting the people of all nations!* To prepare a suitable edifice for this philanthropic union, he began to pull down his chateau; but being interrupted by his friends, he came to Paris, and one day jumped off the Pont-Neuf into the middle of the Seine. He swam manfully and reached the shore in safety. He was so proud of this exploit that he considered himself invulnerable, and began next day, to run in the way of every carriage or fiacre he met in the street, calling to the drivers that they need not mind him, as he could not be injured. He was seized and carried home, but in a day or two, jumped out of his chamber window into the street. He was then put under M. Esquirol, and now remains an incurable maniac in Dr. E's establishment.

The curious case of Louvat, a shoe-maker in Venice, published by Dr. Marc, illustrates the melancholy fate of our illustrious countryman, the late Mr. Whitbread. Louvat, under some strange religious, or rather fanatical impression, cut off the genital organs, and threw them out of the window of his apartment into the street, having every thing ready to dress the wound, from which he recovered without any accident. Soon after this, he became persuaded that God had ordered him to die on the cross. After two years meditation and preparation, he actually nailed himself on a cross, being crowned with thorns, and contrived, thus, to suspend himself on the side of his house. He was taken down in the morning and carried to the public hospital, where he recovered of his wounds, but not of his hallucination. Being confined in a lunatic asylum, he ultimately died of phthisis.

To the disgrace of human reason, it must be acknowledged that, in all ages and nations, abused religion and superstitious creeds have led man to lift his hand against his own life. History informs us that, when the doctrine of the immortality of the soul issued from the Platonic school and became disseminated through Greece, numbers of people, discontented with their condition in this world, committed suicide, in the hope of enjoying an incorporeal beatitude in another world. It is probable, that Christianity would very generally tend to produce the same effect, were it not that patience and resignation under the ills of life, are continually inculcated, and the non-observance of them threatened with future punishment. In this way, all possible excuse is taken away from the suicide, and no person in his right reason, and who believes in the tenets of Christianity, can entertain an idea of self-murder.* We have made use of the terms "right reason," because we are convinced, that lunacies and suicides apparently resulting from religion, do really arise from a *misconception* or *misconstruction* of scripture, which misconceptions or misconstructions working on weak minds, derange the intellect, and, in that way, lead to a lunatic asylum or an unnatural death. Religion is not, therefore, accountable for these imbecilities of the human mind. It is the abuse of religion, the same as it is the abuse of love, which leads to insanity and suicide. Of all religions, Christianity has been the most effectual, indeed, in bridling the passions of man, and thus preventing the crime under consideration. The proof of this may be seen in the tremendous impulse to suicide which scepticism and irreligion have generated in this country, as well as on the Continent. Well may Dr. Falret say:—

> L'irreligion est certainement une cause tres frequente de suicide. Celui qui pense que l'homme meurt tout entier, qui ne croit pas à une autre vie, est necessairement disposé à abandonner celle qui lui paraît une source de calamités."

Scepticism, or the *doubt* of an hereafter, must very generally lead to the same thing as downright *disbelief*—for, as Dr. Falret justly observes, "of what weight in the balance will be the *doubt* of futurity against the *reality* of present misery?"—The author in question has recorded the case of a youth (we might say a child) of 12 years of age, who hanged himself because he was justly corrected for a fault, first leaving a written paper behind him, containing all manner of blasphemies against every thing held sacred by mankind! What must be the system of education or the state of morals

---

* It is curious that the crime of self-murder is not distinctly named or denounced in the Evangelists. But there is abundant collateral proof that the act of suicide was condemned by Christ and his disciples.

in a country, where a youth of that tender age could be embued with such sentiments and principles!

It is unnecessary, we think, to review the influence of the different sects and religions scattered over the earth, on the passions and tendencies of mankind. As we have before said, there is no religion so influential as the Christian, in restraining the passions and checking the crime of suicide. Dr. Falret conceives, that the form of government must exert considerable influence on the tendency in question—and, indeed, on mental diseases in general. Under a despotic government, which checks the expression of the passions, there are few insane, and few suicides—excepting during those dreadful crises when one tyrant, or set of tyrants, dispossess another, involving a number of people in the struggle. There are some exceptions, however, to this rule respecting despotisms. Thus, the Japanese, who groan under the most cruel of all tyrannies, despise death, and rip open their bellies on the most trifling occasions. At this we need not be surprized, when we reflect that, in that country, almost all crimes, without distinction, are visited with death. The constant spectacles of capital punishment must, therefore, render the mind callous to death, in fear or expectation of which, the people must constantly live.

The "esprit militaire," our author thinks, may have a tendency to increase suicide, by inculcating the despisal of death. The Roman soldiery, at one epoch, were very much addicted to this crime—partly in order not to survive the disgrace of a defeat—partly to avoid serving in the triumphal march of their conquerors. It is to be observed, however, that very few instances of suicide obtain during active warfare. The disastrous retreats of the French army from Moscow, and of the English army from the interior of Spain, produced, we believe, not a single instance of self-murder. During a campaign indeed, the soldier is too much employed in fighting the battles of his country, and has too many opportunities of shedding his blood in an honorable manner, to think of suicide. Suicide, like duelling, is principally confined to garrisons, and to times of peace, when the passions, which were kept in check by the pursuits of ambition and of glory, burst forth in these ugly and disgraceful shapes.

Republican forms of government are those, which give greatest latitude to the turbulent passions of mankind, and, thus, favour the development of mental alienation, and consequently suicide. It is curious, that it is not immediately during the explosion of political revolutions that these two diseases of the mind appear—but, immediately before, and for some time after these commotions. The reason it is not difficult to explain. While the revolutionary storm lasts, people are too anxious for their own lives and those of their friends, to commit self-murder. But when a political catastrophe of this kind is on the eve of taking place, the imagination magnifies the danger and the consequences, and the tenor of the human mind often gives way during the conflicting emotions engendered by suspense. After the conflict is over, people have time to reflect on the losses they have sustained in friends and fortune, and then insanity and suicide are prodigiously augmented. The French revolution exhibited numerous illustrations of these remarks.

It is abundantly evident, that the progress of civilization must be attended with a corresponding increase of mental as well as corporeal maladies—and, particularly, from over-excitement of both systems. Thus we find, that suicide, so common in England, France, and Germany, is almost unknown throughout those vast countries under the dominion of the Czars.* It is somewhat remarkable, that Switzerland appears to be freer from this crime than any country in Europe.

The tendency to suicide is always in relation to the state of public manners. In examining the history of the Jews we find this crime nearly unknown while they continued submissive to the laws, faithful to their religion, and animated with the love of their country—but suicide became frequent, when they were divided by schisms and torn by factions. The horrible spectacles of self-destruction exhibited by the Jews at the siege of Jerusalem, have been handed down in the page of history.

In respect to Greece, it was remarked by Atheneus, that when the daughters of Miletus put themselves to death, the Milesians had then passed from a state of the greatest fortitude to one of the greatest effeminacy.

From the battle of Pharsalia, (the tomb of Republican Rome) may be dated the frequency of suicide among the Romans. The succeeding epochs of tyranny and cruelty exhibited by their emperors, and the change of manners in the people, were well calculated to call forth the most melancholy and mortifying reflections among all who were capable

* M. Chevrey, in his Medical Essay on Suicide, states that, when a captive in Russia, on the border of the Caspian Sea, he one day made enquiries of a very intelligent peasant respecting suicide. The peasant was utterly astonished when he was told that, in M. Chevrey's country, people frequently destroyed themselves. After sometime he observed that, of course, they must be insane when they committed such unnatural acts.

of thinking, and of contrasting these wretched times, with the splendid æras of Rome in its republican career of glory and heroism. The hosts of pimps and informers under the degenerate and despotic emperors, rendered private, as well as public life a scene of terror and uncertainty, and led to frequent suicide in order to avoid the frightful tortures which awaited the victims of tyranny and oppression.*

Some of our author's modes of accounting for the rise, progress, and increase of suicide in England, are not very satisfactory. It was, he thinks, in the reign of our eighth Henry, when religious schisms began to distract men's minds, that this frightful propensity first began to make head in this country. In later periods of our history, the discussions carried on respecting the question, whether man has or has not a right to free himself from existence when he pleases, (and on which question we shall make some remarks farther on) contributed, no doubt, to increase the crime, in consequence of the various arguments brought forward in its defence. These arguments would doubtless make a much greater impression on the *wavering* mind, than their opposites, and give an additional impulse to all those in whom the suicidal tendency had begun to operate. In our own days, however, there can be no doubt that the frequency of suicide among us, is attributable to a great number of causes, among which, we may particularly distinguish—commercial speculations, political contentions, fanatical extravaganzas, scepticism and irreligion, (which ought probably to have been put at the head of the list) idleness and extravagance, among the upper classes, drunkenness and debauchery, among the lower—and, may we add, the extreme sensibility to public opinion among all classes.

The operative causes of suicide in France, at the present day, are rather tenderly touched on by both our authors;—partly, we suppose, from the restrictions under which the liberty of the press exists in that country, but principally, we imagine, from that propensity which every Frenchman exhibits, (a propensity more amiable than philosophic) of drawing a veil over the failings of his countrymen. We think the following sketch, however, pretty correct; and sufficiently comprehensive to include the principal, or most of the principal causes of suicide, on the other side of the channel.

---

* To cite but a single example:—Vibulenus Agrippa, a Roman knight, while his accusers were stating their fabrications in the senate-house, swallowed poison in the midst of the senators. The lictors instantly seized him, and dragged him in the most barbarous manner into prison, where they quickly commenced their tortures, which were continued for some time after life had departed from their victim!

"Independently of the general causes which have already been alluded to, if we cast an eye on the actual situation of our own country (France)—on the numerous projects disconcerted, hopes annihilated, hands without employment, families derobed of their ancient splendour, and pretensions unsatisfied; we cannot wonder at the quantum of discontent which is now operating. What difficulty in striking out new roads to prosperity, or even to procure the means of existence! How hard is it to languish in an ignoble repose that forms such a contrast with the tumultuous agitation and excitement of our late existence! Can men accustomed to cut their way to fortune (violenter la fortune) be satisfied with domestic quietude, and the humble rank of citizen? In fine, the state of inertia which has now succeeded to enterprises the most perilous, to labours the most Herculean;—the violent shocks of opposing interests;—the animosity of contending parties;—the clashings of sentiment among families the most intimately allied;—the increase of luxury and artificial wants;—the instability of social institutions; and finally, the fluctuation of public opinion—these are the veritable causes of suicide in France."*

Dr. Falret adds, by way of codicil to the above picture, what we consider to be the most important trait of all—namely, that the multiplied and too powerful impressions which have so long operated on a people naturally irritable, have produced a deep taint in their physical constitution. The sensual passions take up their residence in the most effeminate bodies, and they excite the greater irritation as the means of satisfying them are wanting. These privations form the torment of existence, and produce a disgust of life.

"Ajoutons que des impressions excessives et multipliées chez un peuple naturellement si irritable, ont porté une atteinte profonde à sa constitution physique. Toutes les passions sensualles logent dans des corps effeminés; ils s'en irritent d'autant plus qu'ils peuvent moins les contenter. Cette privation fait le tournent de l'existence, et l'on se sent porté au degout de la vie, &c."

We shall not enter into the discussions respecting the comparative frequency of suicide in Paris and London. Drs. Esquirol and Falret have questioned the statements of our countryman, Dr. Burrows, on this point; and he has given a satisfactory reply in a late number of the Medical Repository, to which source we refer those who are curious on this subject. It is sufficient to say that this rash and unhappy practice is too prevalent on both sides of the Channel. It appears that in the year 1817 there were 351 suicides in Paris and vicinity, and in 1818 the number was 330—a frightful catalogue of self-murders.

---

* Falret, p. 94.

Dr. Esquirol (from whom, by the bye, Dr. Falret has taken much of his information on the subject of suicide) makes many curious and important remarks on the distinction between distaste, or, as it is termed, *ennui* of life, and what may be called *hatred* of existence. This last he considers as an *active* state. It supposes a kind of irritation or morbid sensibility. Ennui, on the other hand, is a *passive* state, the product of an atonic condition of sensibility. Hatred of life is of frequent occurrence, because a thousand causes tend to produce it—it is the most common source of suicide. It spares no class of society, though it is more commonly seen among those who are surrounded with riches and dignities—because these individuals are more subjected to passions, and to passions of the most violent kind. A prey to real or imaginary ills, man first becomes affected with distaste, then disgust of life, and this too often ends in suicide. When we say distaste or hatred of *life*, we ought rather to say of the *ills* of life—for many suicides did, and still could enjoy life in the highest degree, were it not for the ills they suffer, or think they suffer.

"Man," as Esquirol justly observes, "requires change of impressions, and something to desire, otherwise he sinks into tædium vitæ; but if he exhausts his sensibilities by habituation to emotions too vivid; by the abuse of pleasures;—if, having ransacked every sourse of happiness, he at length finds that none of them are capable of affording further fuel of enjoyment, then all external objects become indifferent—he lives, as it were, in a hideous solitude, and falls into the most wretched state of ennui, which leads to suicide. To quit life is to him an act of as much indifference as to quit a table where he had no appetite."*

This kind of suicide, Dr. Esquirol denominates chronic or "splenetic." It is executed with perfect calmness and sang froid. There is nothing to warn the friends of the impending catastrophe. Its causes are generally those which act by debilitating the nervous system, as libertinism, onanism, intemperance in drink. In common with other suicides, however, they exhibit the same change of character and habits—the same indifference for objects previously the most dear—the same physical symptoms, as loss of appetite, insomnium, constipation, emaciation—the same concentration of attention on a single idea or train of ideas—the same predominance of one particular moral affection—the same integrity of judgment on every other subject—the same ob-

---

* Dict. des Sciences Medicales, art. Suicide, p. 228.

stinacy—the same dissimulation in the execution of their designs.

Dr. Esquirol states that he has often seen a variety of suicide, not noticed by authors, but which, he thinks, bears great analogy to what has been denominated by English writers the "SPLEEN."

"There are individuals," says our author, "who, from various physical or moral causes, fall into a state of corporeal torpor and mental depression. They complain of loss of appetite, dull pain in the head, sense of heat in the stomach and viscera, borborygmi, and constipation of the bowels; while they exhibit little or no external indication of disease. In the female sex, the menses are sometimes suppressed. As the complaint advances, the features alter, and the countenance exhibits anxiety—the complexion becomes pale or sallow—there is a sense of tightness or even pain in the epigastrium; a kind of confusion in the head which prevents them from fixing their attention or arranging their thoughts; a general torpor or lassitude which keeps them inactive. They dislike to move out, and love to loll about on a sofa—they are irritated if you advise them to take exercise—they abandon their ordinary occupations—neglect their domestic concerns—become indifferent to their nearest connexions—in short, they will neither converse, nor study, nor read, nor write, shunning society, and being impatient of the inquiries or importunities of friends. In this state they become filled with gloomy ideas (idées noires)—despair of ever being better—desire and even invoke death, and sometimes destroy themselves, from a conviction that they are no longer capable of fulfilling their duties in society. These people are perfectly sane on all subjects of conversation; their impulse to suicide being strong in proportion to the activity of their former occupations, and the importance of their former duties. I have seen this disease (for it is a disease) continue for several months; for two years. I have seen it alternate with mania, and with perfect health. I have seen patients who would be six months of the year maniacal or in sound health, and the other six months tormented with these gloomy ideas and impulse to suicide."—*Dict. des Sciences Med.* v. 53.

Here Dr. Esquirol relates some curious and interesting cases illustrative of the foregoing description, of which we can only notice one or two. One was a gentleman 32 years of age, of apparently good constitution, having never experienced illness, and who had had a good education, but adapted to a lucrative business for which he was designed. He was married to a woman whom he loved at the age of 27. Some affairs went wrong with him a few years after marriage, which greatly discouraged him, and rendered him inactive, without apparently affecting his health. He now embarked in a speculation which promised much advantage, and at first applied himself to business with unremitting assiduity. In the course of a month, he encountered some difficulties, which depressed him beyond measure. He considered himself ruined—refused to quit his bed, and would not superintend his workmen, from a conviction that he was no longer capable of directing their operations. He complained of head-aches, heat in his stomach and bowels, and constipation. His affection for his wife and children, his pecuniary interest, all failed to rouse him from this prostration, moral and physical. He reasoned sanely on the critical situation of his affairs, and yet made no effort to extricate himself from his difficulties. Eight days passed in this way ; when, all at once, he sprung from his bed in perfect integrity of mind and body. He resumed instantaneously all his activity for business, all his affection for his family. The same state, however, recurred ten or twelve times since, at irregular intervals, caused, in general, by trifling contrarieties of business which, under other circumstances, would be considered as nothing. During several of these paroxysms he has had impulses to suicide; but this dreaded catastrophe has not yet taken place.

A remarkable case is also related of a female, who entered the Salpetriere on the 23d of September, 1819, in the 34th year of her age, and fourteen years after marriage. At the age of 21 she had a child, after which she was affected with an ulcer in the foot, which was healed in six months. From this time she was troubled with cardialgia, at first slight, but afterwards with intense pain and vomiting of her food. These symptoms continued more or less till her second pregnancy, at the age of 27, when the stomach affection became greatly exasperated, and she was convinced there was cancer of that organ. At the age of 33 she became irresolute in her ideas and actions, with dislikes to those things she before was pleased with, and occasional incoherencies. In the course of six months she became sleepless, and complained of pain at the root of the nose. Her face assumed a pallid aspect, the features altered, and the countenance exhibited something wild. There was pain in the stomach, and sense of tightness in the epigastrium. She now abandoned her household affairs, became greatly dispondent, and tried more than once to commit suicide. In this state she came into the Salpetriere, and was put upon diluents, low diet, and the tepid bath. In three months the mind became more composed, and she wished to have some employment. But the sense of tightness in the epigastrium and the sleeplessness continued obstinate. She was put on aperient whey diet, and had a blister to the nape of the neck. This last produced great irritation, and the whey brought down copious stools. She now began to have sleep; hope revived in her breast, and she worked with pleasure. She was able to return to her family on the 23d March, 1820, but has since had several threatenings of relapse, and at the time of writing, this unhappy patient was harrassed with gloomy ideas, despair of recovery, and desire to quit a life, the duties of which, she no longer found herself able to fulfil.

"But it will be said (continues our author) that there are individuals who, in the midst of affluence, grandeur, and pleasures, and in the full enjoyment of reason, have suddenly put an end to their existence, immediately after parting with their friends in good spirits, or after having written letters on business with perfect correctness—can these be said to be insane when they commit suicide? yes, most undoubtedly. Do not monomaniacs appear perfectly sane on all other subjects till the particular idea is started which forms the burthen of their hallucination? Are they not capable of curbing the expression of their delirium, and dissembling their aberration of intellect? It is the same with some individuals over whom the suicidal idea tyrannizes. A physical pain, an unexpected impression, a moral affection, a recollection, an indiscreet proposition, the perusal of a passage in writing, will occasionally revive the thought and provoke the act of suicide, although the individual appears the instant before in perfect integrity of mind and body."—*Esquirol ut supra.*

Pinel relates an analogous case of a maniac confined in the Bicetre at the breaking out of the revolution, and who, appearing perfectly sane when the revolutionists were forcing open the various prisons, was paraded about in triumph as one of the supposed victims of regal tyranny. Before the triumphal procession was closed, however, the tune was turned; for, excited by the vociferations and military spectacles around him, the original homicidal hallucination was called up—and he sprung among the people, sword in hand, aiming at the life of every one within his reach!

M. Esquirol has never seen an unequivocal instance of any individual drawn to the commission of suicide by a kind of irresistible impulse, as it were, and independent of any secret grievance, real or supposed. We have only seen one instance (a female) where this impulse is confessed, and is described as perfectly involuntary and irresistible. She owns to no source of grievance, except this unfortunate propensity, which is periodical, returning almost every day circa meridiem, after which she feels nothing but a horror at the propensity just vanished. This lady is not under our own immediate care at present; but we had her history from her own mouth about nine months ago. We are inclined, however, to agree in a great measure, with the opinion of M. Esquirol, that could the secret feelings of these suicides be accurately ascertained, there would generally, if not always, be found some lurking source of discontent, real or fanciful, in the breast, which served as motives to this suicidal propensity. Many instances are on record, it is true, where men have put a period to their existence without any visible cause or motive; but, as Rousseau has justly observed—" le bonheur n'a point d'enseigne exterieure: pour en juger, il faudrait lire dans le cœur de l'homme heureux."

" Individuals," says M. Esquirol, " who appear outwardly the residence of happiness, are often inwardly a focus of chagrin, and tortured with distracting passions. That man who destroyed his own life, being at the same time, happy in his mind, is a phenomenon which human reason cannot comprehend."

It is a curious feature in the history of suicide, that it is sometimes preceded by a tendency to homicide. M. Esquirol observes, that all the instances of this kind which have come before the public, have a surprising analogy with one another. They appear all to have laboured under monomania, or an hallucination on a particular topic, being perfectly sane in all other respects. They generally choose for victims those who are, or were, most dear to their hearts. They commit the homicide with an apparent calmness and tranquillity, which are not disturbed when the horrid act is perpetrated. On the contrary, they often appear gratified in having accomplished their object. Many have gone immediately to the proper authorities and confessed their crime, or disclosed it to the first of their acquaintances whom they met. Far from concealing the transaction, they usually give themselves up to the officers of justice, and demand the capital punishment attached to murder.

Sir A. Chrichton, in his able work on mental derangement, relates several cases of suicidal homicides. These unfortunate beings not having the courage to destroy themselves, committed homicide on others, with the view of expiating their crimes on the scaffold. The examples are extremely numerous of individuals who, in a paroxysm of anger or jealousy, have slain the object of their passion or vengeance, and then plunged the dagger in their own breasts. The following case, we think, cannot easily be paralleled. It is related by Gall in his physiology of the brain. The first lieutenant of a company in which Prochaska served became enamoured of the wife of the latter; but she resisted all his entreaties. The officer, irritated by this obstinacy, was guilty of some injustice to the husband. Prochaska appeared dejected and morose; but the following day he dined as usual, and seemed quite tranquil. On the fourth, both he and his wife confessed, and took the sacrament. He dined in good spirits, and took a few glasses of wine. In the evening he and his wife went out to walk, and he expressed himself in terms of great affection for her. He asked her, however, if she had made a candid and full confession to the priest; and on being answered in the affirmative, he plunged a poignard in her breast. Seeing that she was not instantly dispatched, he cut her throat across, in order to more quickly release her from her sufferings! He now repaired to his home, and seizing his two children, who were in bed asleep, he actually hacked them in pieces with a hatchet. These three murders committed, he repaired to the main guard, and, with the most perfect coolness and deliberation, detailed the whole particulars of the bloody deed. He concluded in these words:—"*Let the lieutenant now make love to my wife if he pleases.*" We are forced to accord with our intelligent author, M. Esquirol, in the following sentiment:—

"Can we believe that such a violation of the first law of Nature, that such an excitement of the imagination, that such an estrangement of the sensibility can be reconciled with the plenitude of health and the enjoyment of reason? Must not, on the contrary, that being have arrived at the highest pitch of delirium, who assassinates the wife whom he cherishes and the children whom he loves? Yet numerous facts prove that these unhappy wretches, before and after the execution of these acts, and in all things else but these acts, were perfectly sane. This sanity, however, is the sanity of the madman in his lucid intervals, which is liable to be instantly dissipated the moment that the hallucinatory idea crosses his mind. It is not that the symptoms or signs of delirium are wanting in those who commit suicide—but that there are wanting close observers to detect them." *Esquirol, p.* 239.

The instances of reciprocal suicide on record are not rare, both in ancient and modern times. The melancholy fate of Arria and Pœtus is well known to the reader of Roman History. Pœtus being condemned to death by the Emperor Claudian, his wife Arria disdaining to survive her husband, plunged a dagger into her breast, and drawing forth the reeking weapon presented it to Pœtus for a similar purpose. In our own country, and in the year 1726, Richard Smith, his wife, and their infant, were all found dead. They first destroyed their child, and then hanged themselves to the bed posts. They left the following sentence in writing on their table:—"We believe that God will forgive us. We have quitted this life because we were miserable and without resources; and we have put to death our infant to prevent him becoming miserable also." Such coolness and collectedness would appear to preclude the idea of insanity; but we think the following fact will shew that mental derangement was at the bottom of this horrid transaction. At the same time that Smith and his wife murdered their only child, and wrote the foregoing explanation, they also wrote to a particular friend of theirs, recommending to his special protection their dog and cat!

"In the year 1770 a fine young man at Lyons became deeply in love with a young lady of the same place, whose parents refused consent to their marriage. The young man burst a blood-vessel by accident, and the physicians gave it as their opinion that he could not recover. This being known, the two lovers contrived to meet, the lady bringing with her a brace of loaded pistols, and a couple of daggers. They embraced for the last time. They both fired at the same instant, and both fell dead on the ground! This example became famous, and unfortunately has been too often imitated since." *Esquirol.*

Suicide is sometimes feigned, especially by hypochondriacs and monomaniacs, for the purpose of obtaining some object, or harrassing and annoying their friends. They talk a great deal about putting themselves to death, and when they do make any attempt, they take good care that they shall be surprised, or rather interrupted in the act. If not, they are feeble and abortive aims at their own life.

M. Esquirol doubts whether there be many suicides who are driven to this act by an irresistible impulse, and without design, without previous reflection. On questioning a great many people who had made attempts on their own lives, they almost all declared that they had gone voluntarily to the act—and not only so, but with great satisfaction, as to the goal of their earthly career, now a burden to them. Nor is it true that they, in general, come to these determinations, without much previous balancing and internal distraction of mind, as we shall see further on in detailing some remarkable instances.

We now return to Dr. Falret. This physician observes that, although suicides present an infinite variety among themselves, yet, considered in a general manner, the suicidal aberration of mind assumes two principal and opposite forms—the one characterized by a profoundly concentrated despondency and love of solitude—the other by a strong excitation, physical and moral. This last species of suicide generally takes place suddenly at the winding up of some great gust of passion, the symptoms accompanying it being as variable as the passions themselves. In such cases, the act generally succeeds the resolution so quickly that the physician is only called in to witness the catastrophe. In other cases, the march of the aberration is more slow, and the attentive observer may often seize the traits and prevent the consequences. The countenance assumes a very great variety of expression, with some convulsive twitchings occasionally—there is more colour than natural in the face, with general symptoms of determination to the head; cephalalgia; insomnium; and great variations in the sensibility.

Some melancholics assert that they have experienced an inexpressible anxiety for some time before they attempted their lives. They felt their heads in a state of dreadful confusion, and sought self-destruction as much for the purpose of freeing themselves from their present sufferings, as in consequence of the original idea that haunted their minds. Others, on the contrary, have experienced a kind of beatitude, and have rushed on death as the secure harbour against every tempest.

Independently of these symptoms of cerebral affection, there are frequently marks of disturbed function in the abdominal or thoracic viscera—particularly of the stomach. The hypochondria are often tense, and the seat of a greater heat than natural. The action of the heart is generally above par and tumultuous; the respiration being, of course, in accordance. The march of the suicidal delirium, with excitement, is infinitely more rapid than that with despondency—and the prognosis is more favourable, because the desire of self-destruction, if it does not degenerate into insanity, will usually subside with the cause that has produced it. It is to be remembered, however, that this form readily assumes the intermittent type, is susceptible of renewal by the slightest causes, and is likely to become habitual after a succession of attacks. Our author coincides with Dr. Esquirol, that whatever *sang froid* may appear in the act of suicide when once determined on, there is always a great struggle in the human breast before this horrible resolution can be adopted.

It is in vain to discuss the question, in this place, whether suicide be an act of courage or cowardice. In a *medical* point of view the act is the result of intellectual aberration, whether produced by a gust of passion or a slow process of melancholy. It does not therefore fall under the heads of either fortitude or pusillanimity. Some *moral* reflections will be made hereafter, especially as they regard hypochondriacs who are susceptible of reasoning and arguments occasionally, and who may sometimes profit by the judicious *moral* advice of the medical attendant.

The chronic form, or that accompanied by great despondency, is often the ultimate stage of melancholia. It is not at once, but by a long train of perverted reasoning that man works himself up to this unnatural act. These individuals become taciturn, morose, pusillanimous, distrustful. The future presents itself to their view under the most lugubrious aspect, and despair gradually becomes painted in their countenances. Their eyes become hollow, and tinted yellow or somewhat blood-shot—there is cephalalgia—pulsation in the head—sleeplessness, or sleep disturbed by frightful dreams—inappetency—constipation, with hard and unnatural fæces. It is in this state that they hatch their projects of suicide, and the diaries which some of them have kept of their sensations and thoughts, disclose the various kinds of death which they have contemplated and rejected, one after another, often for reasons the most preposterous or ridiculous. It is remarkable that, in these journals, they generally endeavour to hide their despondency and their mental aberration, while their moral and intellectual weakness is sure to be betrayed. They sometimes accuse themselves of insanity, and bewail their unhappy lot—at others, they argue forcibly and ingeniously in favour of their meditated suicide. Others again, subdued, as it were, by the force of the moral and religious principles which they had imbibed, represent to themselves that the act which they contemplate is contrary to the moral end for which man was created—fatal to the welfare and happiness of their families. Then ensues a conflict in their breasts. If reason and religion prevail, the project is adjourned—sometimes even abandoned altogether. If otherwise, the suicide is committed. Our author knew a woman with this tendency, but who had strong and proper principles of religion: It was a long time before she could reconcile herself to the act of suicide, and then it was upon the following ground of sophistry :—"There are no general rules, said she, without exceptions, and I am the precise exception in this case—therefore I may commit suicide without violating my religious principles."

Some people, having once conceived the idea of self-murder, are so horrified that they hasten to perform the act, in order to get rid of the terrible state of anticipation. Others appear to meditate for years on the bloody deed. Of this class was Rousseau. After drawing a piteous portrait of his proscribed and solitary condition, and of the state of his health, he adds, "Puisque mon corps n'est plus pour moi qu'un embarras, un obstacle à mon repos, cherchons donc à m'en degager le plus tôt que je pourrai." He poisoned himself by taking arsenic in coffee!

It has been supposed by some authors that the physical and moral feelings of the individual have great influence in determining the *kind* of death by which they free themselves from sufferings. But this, Dr. Falret thinks, is not verified by experience and observation. In general these unfortunate beings choose that mode of suicide which appears the most prompt and the least painful. Some, however, think little about the means, their minds being too much occupied about the end.

The symptoms of suicide usually go on augmenting till the fatal event takes place; and after the individuals have once come to a fixed determination, their subtlety and address in eluding the attention of their friends and disguising their own feelings is astonishing, and ought to keep all concerned on their guard. This is no proof of the integrity of their intellects, as some have adduced. Those who are acquainted with the cunning and duplicity of maniacs will agree with us on this point.

There is reason to believe that the suicidal propensity has sometimes assumed a kind of epidemic character. We have already alluded to this event, as recorded by Plutarch, among the women of Miletus. Montaigne asserts that the Milanese, during a time of war, were affected in this way; and the same observation has been made by many historians and physicians. In the year 1806, more than 60 people destroyed themselves in the city of Rouen alone, during the months of June and July. The air had been very moist and hot at this time, and some considerable failures had involved in ruin a great number of individuals. In the same months of the same year, there were more than 300 suicides at Copenhagen, where commercial distresses were severely felt in consequence of what was called the "continental system," enforced by Bonaparte. The year 1793 presented the dreadful spectacle of 1300 suicides in Versailles—occasioned, we need not say, by the dreadful political convulsions of that memorable period.

Viewing the suicidal tendency as a corporeal disease, whether produced by moral or physical causes, it next becomes a question, in what organ or part is the disease situated? The celebrated Avenbrugger and several others assigned to the hypochondria the seat of the complaint, as many have done with respect to mania. Esquirol seems inclined to attribute the suicidal tendency to derangement of the biliary organs, at least in many instances, as would appear from the following among other passages. "Le passage d'un été sec à un automne humide est plus favorable au developpement des affections abdominales, *dont le suicide depend si souvent.*" —*Art. Suicide.* M. Falret does not coincide with this last, nor with any of the authors who have written on suicide. He conceives that the disease can have no other seat than the brain, seeing that it is always accompanied by derangement of the intellectual faculties. That the organ of thought is invariably affected, whether in function or structure, in people who commit suicide, we can entertain little doubt; but then the question occurs, is the brain primarily or sympathetically affected? M. Falret is of opinion that it is *almost always* primitively affected, and that lesion of the other organs is rarely the cause of the cerebral disorder which leads to suicide. We only differ from our author on the last point. We think the head is more frequently affected in a *secondary* manner than M. Falret imagines. We agree with him, however, on one point, namely, that moral causes predominate far over physical, in the production of suicide, as well as insanity in general. Yet still these moral causes—grief, we will take as an example, derange various other organs as well as the intellectual functions, and these derangements react on the sensorium, and contribute still farther to the cerebral disturbance. We shall not enter, however, into the controversy between M. Falret and his antagonists respecting the *seat* of suicidal alienation. The *immediate* seat must, of course, be the brain—unless it can be proved, that the organ of thought is in the thorax or abdomen. The *causes* of the intellectual disturbance may be, as we before observed, at a distance from the sensorium. By confounding the cause with the effect, all this discussion has been originated, and, therefore, we shall pass it over entirely.

In respect to the treatment of this species of insanity, little or nothing can be added to what is known of the treatment as regards mental derangement generally. All observers have dwelt much on the utility of exercise; and M. Pinel's wish, that a large enclosure were attached to every lunatic asylum where the patients might have the benefit of agricultural, or, at least, of horticultural employment, is likely to be realized by the new Commission for Lunatics in France. Horse and carriage exercise, as well as foot, is very beneficial. In all cases where there is excitement, and this almost always obtains at the beginning of the disease, local or general depletion will be necessary—warm baths to the lower parts of the body, and cold applications to the head—aperient and alterative medicines, and all such as tend to restore whatever function appears to suffer most.

"But what treatment can we employ in cases of *spleen?*" Or where can we find enjoyments for the man who has exhausted the whole round of pleasures? The advice which Fenelon makes the shade of Diogenes offer to the tyrant Dionysius, may here be given.—"To procure for yourself an appetite, you must suffer hunger—to dispel the ennui of your gilded palaces, you must occupy the tub which I have lately vacated in the other world." Isolation is as necessary, M. Falret thinks, in suicidal, as in other kinds of mental alienation. In general, he avers, it is not of much use to enter into long reasonings or arguments with these people. It is proper to leave them rather abruptly, after saying something abiguous that may excite reflexions in the physician's absence. A little flattery, he thinks, is sometimes useful, and helps to draw from them the secret of their chagrin. "It is often of advantage to exaggerate the happiness that awaits them on their discharge from the asylum. Illusions are the opiates of moral sufferings; and is not this the time to administer them unsparingly, when they are the only means left of rendering life supportable?"—It is curious, that a repetition of the same cause (to a greater extent) that produced the suicidal tendency, has removed the affection altogether. Thus, Professor Moreau relates the case of a lady, who lost a considerable part of her fortune, and became suicidally melancholy. Sometime afterwards, the remainder of her fortune was lost also, and now she was cured by being obliged to procure the means of subsistence by manual exertions.

Vivid and unexpected impressions have frequently turned the suicide from his desperate attempt, and even cured him altogether. The history of our countryman who was attacked by two footpads on his way down to the Thames to drown himself, is well known. He lived to the age of 80, and never afterwards renewed the attempt. Although it is desirable not to shew our suspicions of these unfortunate individuals; yet we must never remit our precautions till the danger is entirely over. These people endeavour to inspire us with confidence in their security, only to deceive us the more effectually, the moment they find a favourable opportunity. Petit has related a case, some particulars of which will be interesting, as bearing on this point. A soldier, who was greatly beloved in his regiment for his exemplary conduct and amiable qualities, became affected with suicidal melancholy, and fired a pistol into his mouth. The havoc made was dreadful; but, by great exertions, on the part of M. Petit, his life was preserved; and as, during the danger, he manifested great anxiety about his own recovery, and great gratitude to his surgeon, the latter entertained every hope that all suicidal tendency was dissipated. The whole was a manœuvre on the part of the patient, who, by this dissimulation, got more liberty than would otherwise have been accorded, and soon found means to put an effectual period to his existence, even while in the ward of the hospital!

M. Falret is correct in stating that, generally speaking, the sight of joyous or gay spectacles irritates the melancholics rather than affords them amusement. He has led people of this description to places of amusement, and afterwards through the wards of hospitals where human misery was sufficiently evident, in order to mark the effects of these contrasting impressions. He always found, that the visits to the scenes of suffering were more useful to the melancholic than the opposite scenes—probably, by suggesting to the patient that his own condition was not the very worst in the world. It is probable that many people think themselves miserable because they are not acquainted with the misery of others. But it would be endless to pursue all the *moral* indications which present themselves in the management of suicides—and indeed of mental derangement in general. It is in the important branch of moral *therapeutics* that the genius and discernment of the physician are more conspicuously displayed, than in the physical treatment of the insane.

We are now to make a few reflections, legal and moral, on suicide. One of the authors before us, M. Falret, considers all laws against suicide as completely useless, as well as unjust, and even dangerous. M. Esquirol seems to be of a different opinion. "Suicide (says he) has become more frequent since the laws which condemn it have lost their vigour. For the interest of society, therefore, the legislator ought to enact laws—not so much for the punishment as the prevention of the crime." He does not hint any particular kind of law, but he instances (we should suppose by way of approval) the present law in Saxony, by which, the body is given up for dissection.

We know that the law of England imposes dishonour on the corpse, and confiscation on the property of the suicide, provided he be in the possession of sound mind at the time the act is committed. As the suicide is now almost invariably, and very properly, pronounced a lunatic by the coroner's jury, the law is a mere dead letter, and, therefore, we cannot at present, judge of the influence which repressive laws might exert in the prevention of the crime. We have no doubt, that the hands of very many hypochondriacal suicides, who are perfectly capable of reasoning upon most subjects, would be arrested by laws which they knew would certainly be put in execution. It is not safe to infer, as M. Falret has done, that because a suicide is not restrained by the ties of love, friendship, religion, or morality, so neither would he be restrained by any law whose operation would bear on his dead body. The history which M. Falret has quoted in another place, of the stop put to suicide among the women of Miletus, by the edict which exposed the naked bodies of the dead in public places, is very much against his own doctrine. M.

Falret has appealed to the same laws which have been enacted in various countries against duelling—all which have been abortive in preventing the crime. True; but here, as in the case of suicide, the laws have slumbered, and, therefore, are no longer dreaded.

We agree, indeed, with Dr. Falret, that penal or severe laws against the dead body, or the property of the suicide, would be, in the great majority of cases, useless, because the greater number commit the act in a state of insanity that renders them deaf to all laws. He says it would be more wise to fortify the minds of men against this act, "by maxims of religion, philosophy, and a just appreciation of the laws of honor." We fear that acts of Parliament will have little influence in inspiring religious or moral sentiments into people's minds at this time of day! We consider the following project as completely chimerical, as any thing in Plato's Republic, or Moore's Utopia.

> "Il faut inspirer à chaque citoyen le gout de son état; *il faut le soustraire à l'empire des passions dont il est le victime; (!!)* il faut s'efforcer d'arreter les progrês du froid égoisme en resserant les liens de la parenté; il faut regulariser l'amour de soi-même." 282.

Yes! if these things could be done by act of Parliament, then, indeed, we should soon have the Millennium so long predicted! In a ship, or a regiment, where the minds as well as the bodies of men are in subjection to the commander, something of this kind might be effected; but, in a community at large, the attempt would certainly be abortive. By this we do not mean to insinuate, that wise laws and good governments may not have great influence on the minds and morals of a people—we only argue, that no laws or edicts can come so home to the breast, as to withdraw men from the influence and operation of human passions.

Dr. Falret thinks, that the narratives of cases of suicide in the public prints, have considerable influence in rendering the act more familiar to people's minds, and, therefore, more readily fallen into. It is with this as with public executions, much may be said pro and con.* We observe, that Sir Walter Scott, in his "Heart of Mid-Lothian," inclines to the opinion, that the "dreadful note of preparation" and

---

* We think it will be allowed by every one who is in the habit of perusing the public papers, that the instances of suicide, since the melancholy death of the Marquess of Londonderry, have been more numerous, by far, than at any period in the memory of any man now living. Yet, excepting the single class of agriculturalists, there is not near so much distress operating now as at many former periods.

the slight of capital punishments, have an effect in deterring from criminal vices, rather than of rendering the human mind callous to the idea of death as the price of crime.

We shall conclude this article with a few, and but a few, moral reflections on the melancholy subject which has long been termed the disgrace of our island, but which is now too prevalent in most countries of Europe. Viewing suicide as generally, we might say always, the act of a mind unsound for the moment, it can hardly, in strict justice, be considered a crime. The criminality, we imagine, consists, principally, in giving way to those passions and reasonings which pave the way to the desperate act. The physician very frequently comes in contact with hypochondriacs who have suicide in contemplation, and who discourse on the subject with great sang froid. These people will reason with great force, and puzzle the medical attendant who has not thought much on the subject. We are convinced, that it is with these in particular, that some good may be done by judicious representations. Where proper principles of religion and morality have been early instilled into the mind, and even *in part* retained, the arguments against suicide are easily supported, and are irresistible: but, unfortunately, the spread of scepticism is now to such an extent, that this, the strongest, chain of argument, will not be listened to. Of what use would it be to represent to the materialist, (at least the modern materialist who disbelieves in the immortality of the soul) that "by continuing in the world, even when life is hateful to ourselves and useless to others, we retain the opportunity of meliorating our condition in a *future state*."* The sceptical hypochondriac answers that, in the first place, he does not admit of a future state—in the second place, as he did not come into this world by his own consent, as a soldier comes into an army, so no just law can compel him to remain here longer than he wishes. It is in vain, in fact, to urge any religious plea for bearing up against mental or physical sufferings, to the consideration of the sceptic. But it may be urged home on these people, that suicide is, at least, a *selfish* act, and cannot lay claim to any portion of fortitude—or, perhaps, of bravery. It is quite evident, that life, to him, is a greater evil than death, and, therefore, regardless of what may be the consequence to others, he seeks that which seems most agreeable to himself. Such an act as that of suicide never can be circumscribed in its influence to the individual alone who commits it. The bad example to the world at

---

* Paley.

large; (for did mankind throw off all horror at this act the foundations of society would be broken up) the stain on the memory of the dead, and, consequently, the stigma on the relations of the deceased; the ingratitude to Nature and fortune, for enjoying the sunshine, but shrinking from the storms, of life—these and many other cogent reasons for setting an example of patience and fortitude under the ills to which all are liable, and from which few escape, ought to be pressed upon the suicidal hypochondriac with all the eloquence and earnestness in the power of the physician.

